# A Novel Sensitive Technique to Detect *ESR1* Hotspot Mutations in Liquid Biopsy Using Switch‐Blocker–Enhanced Targeted Amplification Coupled With Pyrosequencing

**DOI:** 10.1002/cai2.70054

**Published:** 2026-04-06

**Authors:** Yantong Zhou, Wenna Wang, Bo Lan, Chunxiao Li, Jinsong Wang, Ting Wang, Fangzhou Sun, Yan Wang, Haili Qian, Fei Ma

**Affiliations:** ^1^ State Key Laboratory of Molecular Oncology, National Cancer Center/National Clinical Research Center for Cancer/Cancer Hospital Chinese Academy of Medical Sciences and Peking Union Medical College Beijing China; ^2^ Department of Medical Oncology, National Cancer Center/Cancer Hospital Chinese Academy of Medical Sciences and Peking Union Medical College Beijing China; ^3^ McKusick‐Zhang Center for Genetic Medicine, Institute of Basic Medical Sciences & School of Basic Medicine Chinese Academy of Medical Sciences and Peking Union Medical College Beijing China

**Keywords:** breast cancer, endocrine therapy resistance, *ESR1* mutation, liquid biopsy, PCR amplification, pyrosequencing, switch‐blocker

## Abstract

**Background:**

The detection of estrogen receptor 1 (*ESR1*) ligand‐binding domain mutations in circulating tumor DNA (ctDNA) is crucial for guiding therapy in estrogen receptor‐positive metastatic breast cancer. However, widespread clinical adoption of approaches for monitoring drug resistance and guiding treatment decisions is hindered by limitations of current methods regarding sensitivity, cost, and multiplexing capability.

**Methods:**

The application of switch‐blocker technology, which has been patented for detecting *ESR1* hotspot mutations (Y537S/C, D538G, E380Q, and L536H/P), suppresses the amplification of wild‐type alleles while allowing specific amplification of low‐frequency mutant alleles. We used a switch‐blocker to inhibit the amplification of a DNA target approximately 10 base pairs in length (e.g., the switch‐blocker covering codon 536 of *ESR1* targets various variants at positions 536, 537, and 538). Targeted enrichment was achieved by quantitative polymerase chain reaction, followed by pyrosequencing to confirm mutation components. Next‐generation sequencing and Sanger sequencing served as supplementary methods for the verification of results.

**Results:**

The *ESR1*‐targeted DNA assay was validated for feasibility on plasmid circular templates and ctDNA linear templates. In tests using gradient‐diluted *ESR1* plasmid templates, the proportion of L536H mutant copies increased from 0.0015% to 16.89% after targeted amplification, while the proportion of E380Q mutant copies increased from 0.0015% to 1.35%. In ctDNA samples previously analyzed by next‐generation sequencing, the switch‐blocker considerably enriched other mutant copies within the coverage range of the switch element.

**Conclusions:**

This switch‐blocker–enhanced pyrosequencing assay presents a targeted, multiplexed, and accessible approach for detecting *ESR1* hotspot mutations in liquid biopsies. This assay has potential for dynamic monitoring of therapeutic resistance, facilitating timely treatment decisions in advanced breast cancer management.

## Graphical abstract

This study presents a novel switch‐blocker–enhanced pyrosequencing method, which demonstrates high sensitivity in detecting *ESR1* hotspot mutations in liquid biopsies, providing a new tool for dynamic monitoring of drug resistance and making timely treatment decisions in breast cancer patients. (Graphical abstract image was created in BioRender. Zhou, Y. (2026) https://BioRender.com/13eazvp)

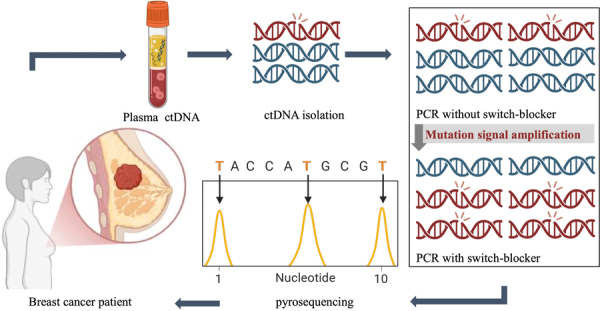

**Abbreviations**
AIs: aromatase inhibitors
CDS: coding sequence
cfDNA: circulating free DNA
ctDNA: circulating tumor DNA
ddPCR: droplet digital PCR
ESR1: estrogen receptor 1
HER2: human epidermal growth factor receptor 2
NGS: next‐generation sequencing
PCR: polymerase chain reaction
qPCR: quantitative PCR
SERDs: selective estrogen receptor degraders
WT: wild‐type

## 1 Introduction

The advent of precision oncology has fundamentally transformed the management of cancer, shifting paradigms toward tailored therapies based on the molecular characteristics of individual tumors. A cornerstone of this approach is the development and integration of liquid biopsy technologies, which enable non‐invasive, real‐time genomic profiling through the analysis of circulating tumor DNA (ctDNA) [1, 2, 3, 4, 5, 6, 7, 8]. Advances in high‐sensitivity, high‐throughput detection technologies based on digital polymerase chain reaction (digital PCR) or next‐generation sequencing (NGS) have enabled the identification of a small fraction of tumor‐derived somatic single‐nucleotide variants, copy number alterations, and structural variants within the large background of circulating free DNA (cfDNA) in blood. These advances have enabled quantitative and qualitative analysis of ctDNA. This capability is particularly critical for cancers characterized by dynamic genomic evolution under therapeutic pressure, such as breast cancer [9, 10].

In breast cancer, estrogen receptor α, encoded by the estrogen receptor 1 (*ESR1*) gene, serves as a primary driver and therapeutic target. Approximately 70%–75% of breast cancers are estrogen receptor‐positive (ER+), and endocrine therapies, including aromatase inhibitors (AIs), selective estrogen receptor modulators, and selective estrogen receptor degraders (SERDs), form the backbone of treatment [11, 12]. Despite initial efficacy, a substantial proportion of patients develop acquired resistance. A key mechanism underlying this resistance is the emergence of activating mutations in the ligand‐binding domain of *ESR1*, such as Y537S, Y537N, Y537C, and D538G [12, 13]. These mutations confer ligand‐independent receptor activity, driving tumor progression even in the presence of endocrine agents. While *ESR1* mutations are rare in treatment‐naïve primary tumors, they are detected in 20%–40% of metastatic, AI‐treated cases, highlighting their role in therapy resistance [14, 15, 16]. The clinical significance of *ESR1* mutations extends beyond the prognosis, which directly informs the therapeutic strategy. The development of next‐generation oral SERDs (e.g., elacestrant, giredestrant, camizestrant, and imlunestrant) has shown promising efficacy specifically in patients harboring these mutations, indicating the need for reliable and repeated mutation profiling to guide treatment selection and monitor emerging resistance [17, 18, 19, 20, 21, 22].

On the basis of the results of the EMERALD study, the FDA approved elacestrant, which is the first oral SERD, for the treatment of patients with ER‐positive, human epidermal growth factor receptor 2 (HER2)‐negative, estrogen receptor α gene (*ESR1*)‐mutated advanced breast cancer who have progressed after previous endocrine therapy [17]. Consequently, the American Society of Clinical Oncology and National Comprehensive Cancer Network guidelines recommend routine testing for *ESR1* mutations in patients with ER+/HER2− metastatic breast cancer following recurrence or progression on endocrine therapy (used alone or in combination with CDK4/6 inhibitors) [23]. Blood‐based ctDNA testing should be the preferred method for this purpose. Therefore, dynamic monitoring of the *ESR1* mutation status by liquid biopsy represents a growing clinical imperative for personalizing therapy in advanced ER+ breast cancer.

Despite these findings, widespread implementation of *ESR1* mutation testing in clinical practice faces considerable technological and practical barriers. The current methodologies present limitations. Sanger sequencing lacks the sensitivity required for detecting low variant allele frequency in ctDNA. NGS, while comprehensive, involves high costs, extended turnaround times, and complex bioinformatics, limiting its utility for rapid, routine testing. Droplet digital PCR (ddPCR) offers excellent analytical sensitivity but is typically restricted to predefined hotspot mutations and requires the design of mutation‐specific probes for each individual variant. These issues result in limited scalability for multiplex detection of co‐occurring mutations or discovery of novel variants. Collectively, these constraints hinder the development of accessible, cost‐effective, and longitudinal *ESR1* mutation monitoring, particularly in resource‐limited clinical settings.

Notably, *ESR1* mutations are characterized by multiple adjacent nucleotide substitutions with diverse mutation types, and detection using conventional fluorescent probe‐based PCR or ddPCR approaches requires the design of distinct probes for each specific mutation. Consequently, comprehensive *ESR1* mutation profiling in a single sample may necessitate the development of more than a dozen probes and multiple parallel reaction systems, substantially increasing assay complexity and cost. In response to these challenges, we sought to investigate a targeted technical strategy aimed at simplifying the reaction system, reducing detection costs, and shortening the turnaround time. During our evaluation of existing mutation detection technologies, we identified the switch‐blocker concept. The switch‐blocker has been previously reported and applied in the context of *EGFR* mutation detection, enabling targeted signal amplification of multiple mutations within a defined genomic region using a single reaction system [24]. This region‐based enrichment capability is particularly advantageous for *ESR1* because of its pattern of clustered, multi‐type mutations within the ligand‐binding domain. By selectively amplifying mutant alleles across multiple adjacent loci, the switch‐blocker approach offers a simplified assay design and enhanced analytical sensitivity. We also aimed to pair this mutant enrichment strategy with a cost‐effective and rapid downstream detection method capable of resolving specific mutation identities. Pyrosequencing, as a quantitative sequencing technology, provides accurate base‐calling with moderate to high throughput and lower operational complexity than NGS. Although pyrosequencing alone is less sensitive than NGS, the substantial signal amplification achieved by the switch‐blocker approach effectively compensates for this limitation, resulting in an overall improvement in detection sensitivity.

Accordingly, this study assessed the feasibility of combining switch‐blocker–mediated enrichment with pyrosequencing for simultaneous detection of multiple clinically relevant ESR1 ligand‐binding domain mutations, including Y537S, Y537C, D538G, E380Q, L536H, and L536P, exploring its utility as a potentially rapid and sensitive strategy for dynamic ESR1 genotyping.

## 2 Materials and Methods

### 2.1 Blood Collection From Patients With Breast Cancer

Whole blood was collected from patients with metastatic breast cancer who were enrolled in prospective studies and a retrospective cohort and were described previously [25, 26, 27]. This study was approved by the Regulatory and Ethics Committees of National Cancer Center/National Clinical Research Center for Cancer/Cancer Hospital, Chinese Academy of Medical Sciences and Peking Union Medical College. In each patient, peripheral blood samples were collected in Streck tubes (Streck, Omaha, NE, USA) and centrifuged within 72 h to separate plasma from peripheral blood cells. The plasma was stored at −80°C immediately after processing to preserve cfDNA integrity.

### 2.2 ctDNA Extraction and Measurement

All clinical samples were derived from patients with metastatic breast cancer enrolled in our previously published prospective and retrospective cohorts. The detailed methodologies for blood collection, processing, cfDNA extraction, and quality control have been rigorously established and described in our previous publications [25, 26, 27]. We isolated ctDNA from plasma using the QIAamp Circulating Nucleic Acid Kit (Qiagen, Hilden, Germany), and lymphocyte‐derived genomic DNA was extracted using the DNeasy Blood and Tissue Kit (Qiagen, Hilden, Germany). A panel of 1021 genes was assayed, and the targeted region covered approximately 1.1 Mb of the 1021 genes including all exons of the *ESR1* gene. All patient‐derived ctDNA samples used in the subsequent ESR1 switch‐blocker feasibility validation experiments were obtained from samples that had passed quality control, based on a comparative analysis with lymphocyte‐derived genomic DNA used as the control.

### 2.3 Template, Primer, and Probe Design and Synthesis

The human *ESR1* coding sequence (CDS) was obtained from the NCBI Gene database. The wild‐type *ESR1* CDS sequence and the 1607T>A mutant sequence were synthesized by GENEWIZ (Suzhou, China). The linear products were ligated into plasmid vectors and verified by sequencing. Primers and target‐activated probes flanking the 1607T>A site were designed on the basis of the *ESR1* sequence. Primer synthesis and probe modification were performed by Sangon Biotech.

### 2.4 Target Selector PCR Assay

Real‐time PCR was conducted primarily using QuantStudio 5 (Thermo Fisher Scientific) and Bio‐Rad quantitative PCR (qPCR) instruments. The qPCR protocol consisted of denaturation, switch‐blocker hybridization, primer annealing, and template extension. Cycling conditions were optimized on the basis of the melting temperatures of the primers and target‐activated probes. High‐fidelity DNA polymerase was used to minimize false mutations during amplification.

### 2.5 Sanger Sequencing, NGS, and Pyrosequencing

PCR amplification was performed using synthesized plasmid DNA and plasma‐derived ctDNA as templates. The products were sent to SinoGenoMax (Beijing, China) and Sangon Biotech (Shanghai, China) for Sanger sequencing and pyrosequencing. In NGS, plasma samples were directly sent to a sequencing company for ctDNA extraction, library preparation, and sequencing.

### 2.6 Workflow of Targeted Sequencing

**Figure 1:**
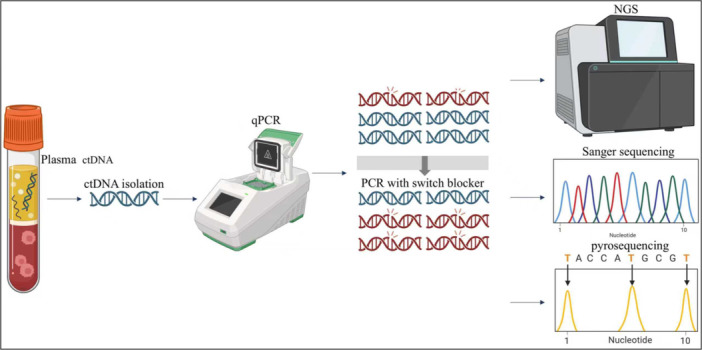
Workflow of targeted sequencing. Circulating tumor DNA (ctDNA) was extracted from plasma, followed by quantitative polymerase chain reaction (qPCR)‐based targeted amplification of mutant sequences using repressive probes. The amplified products were then subjected to qualitative mutation detection using pyrosequencing, and the results were compared with those obtained from next‐generation sequencing (NGS) and Sanger sequencing. Created with BioRender.

Genomic alterations originating from solid tumors are present in peripheral blood as low‐abundance circulating DNA. Accurate detection of low mutant allele frequency variants is critically dependent on assay sensitivity in the context of liquid biopsy. In this study, we developed and optimized a detection method combining a patented switch‐blocker probe with pyrosequencing. This approach selectively suppressed wild‐type DNA amplification while enabling specific enrichment of mutations within the target region. The switch‐blocker strategy facilitated the enrichment of rare variants, and pyrosequencing was used for qualitative assessment of mutation burden. Additionally, NGS and Sanger sequencing were used for validation and a comparative analysis (Figure 1).

## 3 Results

### 3.1 Technical Overview of Switch‐Blockers

The technical approach described in this study is based on a selective amplification system that suppresses wild‐type sequence amplification while allowing mutant sequence amplification by controlling the PCR temperature. To achieve this, a “nucleic acid switch” was designed. During PCR, the switch hybridizes with the target sequence at lower temperatures, separating the fluorophore from the quencher and allowing fluorescence signal detection. Upon switch opening, the fluorophore is brought close to the quencher, resulting in fluorescence quenching, while the target sequence is extended as a template to produce new amplicons. To ensure preferential amplification of mutant over wild‐type sequences, the 12‐nucleotide region of the nucleic acid switch was engineered to have a lower melting temperature when hybridized with the mutant sequence.

**Figure 2:**
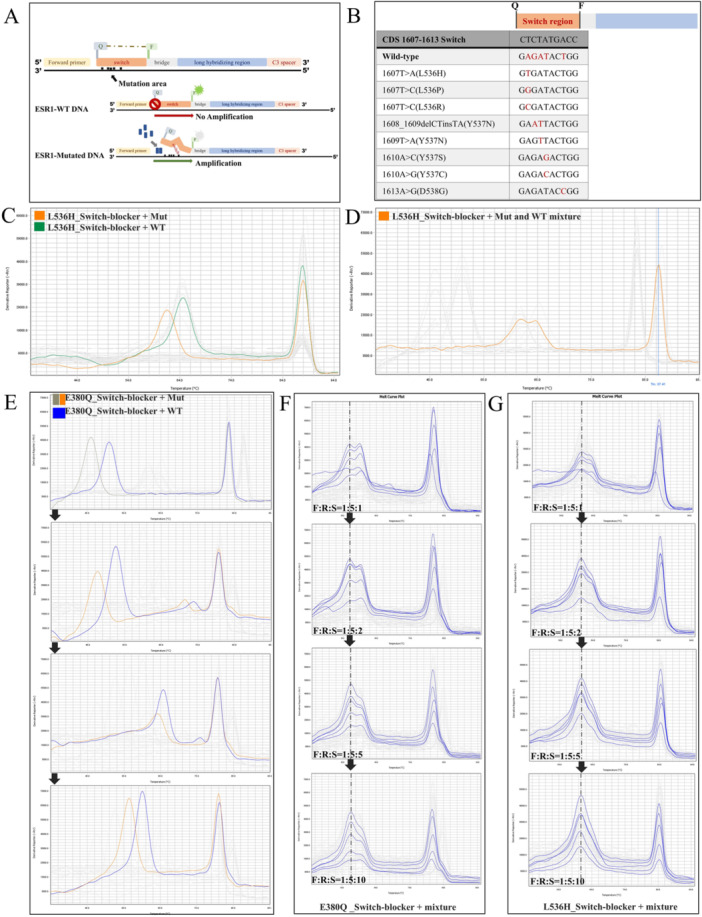
Mechanism and melting temperature analysis of repressive probes. (A) Schematic of the repressive probe mechanism: qPCR amplification is coupled with a proprietary probe to selectively enrich mutant alleles by blocking wild‐type (WT) DNA amplification. (B) Mutation types covered by the switch region of the *ESR1* L536H probe. (C, D) Melting curve analysis of the L536H probe shows clear peak differentiation between mutant (61°C, orange) and WT templates (65°C, green). Mut, mutation. (E) Iterative optimization of the E380Q probe design. Version 1: 46°C versus 51°C; Version 2: 48°C versus 53°C; Version 3: 64°C versus 66°C; Version 4: 56°C versus 60°C for mutant versus WT. (F, G) Increasing the proportion of E380Q and L536H probes in the PCR system enhanced the relative amplification of the corresponding mutant sequences. F, forward primer; R, reverse primer; S, switch‐blocker.

We initially designed a composite switch‐blocker targeting the clinically significant L536H hotspot. The switch region, typically 7–15 nucleotides long, binds perfectly to the wild‐type sequence to block amplification, but forms a mismatch with mutant sequences, leading to destabilization at lower temperatures and enabling amplification of the mutant. A fluorophore and quencher pair was incorporated to signal the “opening” of the switch, and a C3 spacer was added at the 3′ end to prevent extension (Figure 2A). By varying the length and position of the switch relative to the target site, we optimized the melting temperature and identified configurations with superior enrichment performance. The L536H targeting probe also covered adjacent point mutations including D538G (Figure 2B). A melting curve analysis showed distinct fluorescence quenching peaks between the 1607TA mutant and wild‐type templates (Figure 2C,D), and a merged peak was observed in mixed‐template samples. Similar probe design iterations led to the identification of an optimized E380Q probe (Figure 2E). Further optimization of the PCR conditions, including the template amount and probe‐to‐primer ratios, showed that increasing the repressive probe concentration considerably improved amplification of the mutants (Figure 2F,G). Under conditions where the switch‐blocker probe concentration was sufficient to substantially reduce wild‐type amplification, the ratio between upstream and downstream primers also affected the extent of mutant signal amplification. The amplification efficiencies under these conditions were already relatively close. Therefore, pyrosequencing was performed to quantitatively assess differences in mutant signal enrichment. We found that an upstream primer:downstream primer:switch‐blocker probe ratio of 1:5:10 produced the optimal amplification of mutant signals (Table S1). Furthermore, regarding samples with the same mutation type at identical input concentrations, the degree of mutant signal enrichment was consistent, which indicated good intra‐group stability and reproducibility for individual mutation types.

### 3.2 Selection Effect of Switch‐Blockers in PCR Amplification

**Figure 3:**
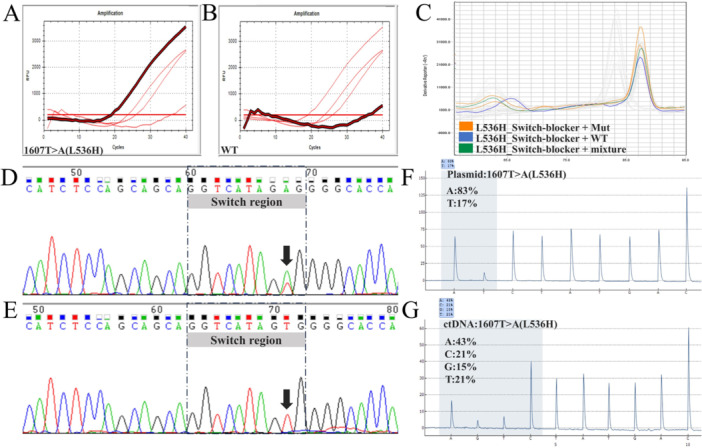
Basic validation of the functional performance of the repressive probe. (A) The repressive probe did not inhibit amplification of the 1607T>A mutant sequence under the specified PCR conditions. (B) The amplification curve of the wild‐type sequence was markedly suppressed by the repressive probe under the set PCR protocol. (C) When the probe was not saturated, a melting curve analysis showed that the product from the mixed template matched the melting temperature of the mutant sequence. (D) Sanger sequencing of amplification products from mixed templates without the repressive probe showed a clear mixture of wild‐type and mutant sequences. (E) When the repressive probe was applied, Sanger sequencing indicated that the amplification product from the mixed template was primarily the mutant sequence. (F) In plasmid‐based mixed templates, pyrosequencing showed an increased proportion of mutant amplification products upon addition of the repressive probe. (G) Pyrosequencing of ctDNA templates showed a marked increase in the proportion of mutant amplicons upon application of the repressive probe.

To conduct controlled tests with known template quantities, template ratios, and template types, we synthesized the *ESR1* wild‐type sequence and, using site‐directed mutagenesis of the wild‐type sequence, obtained known single‐point mutant sequences, which were then cloned into plasmid vectors. Subsequent controlled tests were performed on the basis of these circular vector templates. To preliminarily investigate whether the switch‐blocker could inhibit the amplification of wild‐type sequence templates, we first performed qPCR amplification using templates of the 1607T>A mutant sequence, the wild‐type sequence, and a 1:1 mixture of both. On the basis of the generated melting curves, the extension temperature was set to 50°C, and the concentration of the composite switch‐blocker was adjusted to 10 times that of the forward primer for PCR amplification. The amplification curves were then analyzed. When we compared the amplification curve of the 1607T>A mutant sequence (Figure 3A) with that of the wild‐type sequence (Figure 3B), a clear inhibition of wild‐type sequence amplification was observed. A preliminary analysis of the amplification products via melting curves showed that the melting curve of the mixed sample (wild‐type/mutant sequence = 1/1, Figure 2C) overlapped with that of the 1607T>A mutant sequence (Figure 3C) but was distinct from that of the wild‐type sequence (Figure 3C). This finding indicated that the amplification of the wild‐type fragment was suppressed in the mixed sample, and the 1607T>A mutant sequence product was the dominant species in the PCR products from the mixed template system.

To more definitively assess the effect of the switch‐blocker on amplification products, we performed amplification tests using a 1:1 mixture of the 1607T>A mutant and wild‐type sequences as the template and analyzed the products using Sanger sequencing. In the system without the switch‐blocker, wild‐type and mutant nucleotide types at position 1607 were clearly detectable in the amplification products (Figure 3D). In the system with the switch‐blocker added, only the mutant nucleotide type was detected at position 1607 in the amplification products (Figure 3E). To further precisely evaluate the effect of the switch‐blocker on amplification products, we used pyrosequencing for product analysis. The amplification system containing the switch‐blocker was used to amplify *ESR1* templates from both circular vectors and linear vectors derived from patient plasma ctDNA. After amplification, the proportion of mutant sequences increased from 50% to 83% in the product from the circular vector (Figure 3F), and from 5.8% to 43% in the product from the ctDNA (Figure 3G).

In summary, under the optimized amplification temperature and primer ratio established after testing, the switch‐blocker targeting the 1610 region effectively inhibited the amplification of wild‐type sequences, thereby enabling the targeted amplification of mutant sequences.

### 3.3 The Switch‐Blocker Covers Multiple Mutation Sites and Mutation Types at Consecutive Positions Within the Target Region

**Figure 4:**
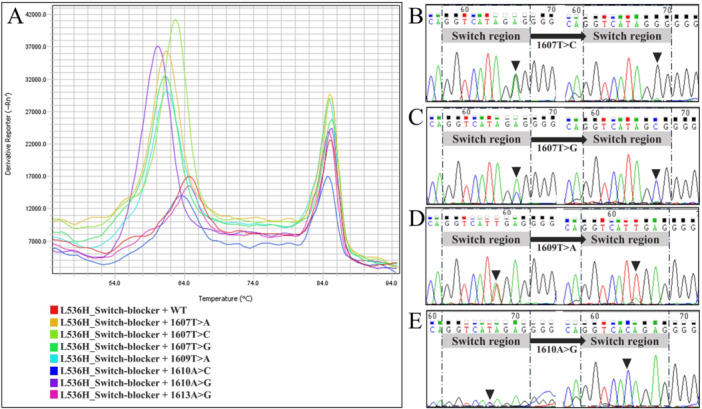
Functional characteristics of the repressive probe at other mutation sites within the region. (A) Melting temperatures of the repressive probe with various other mutant sequences within the region. (B–E) Sanger sequencing of post‐amplification products showed that the repressive probe effectively enabled selective amplification for multiple mutation sites in the region, including 1607T>C, 1607T>G, 1609T>A, and 1610A>G.

To investigate whether the probe has a universal targeted amplification effect on mutant sequences within its coverage region, we performed amplification using templates containing only wild‐type or only mutant sequences. After amplification, the probe was added to generate melting curves. The fluorophore quenching peaks for five point mutations were clearly separated from that of the wild‐type sequence, while the quenching peaks for two other point mutations showed considerable overlap with the wild‐type peak (Figure 4A). Based on the separation of the quenching peaks, the targeted amplification effect may be stronger for the mutations that were more distinctly separated, namely 1607T>A, 1607T>C, 1607T>G, 1609T>A, and 1610A>G. In contrast, the targeted amplification effect for 1610A>C and 1613A>G may be weaker.

To further test the targeted amplification efficiency of the switch‐blocker at different sites, we conducted amplification tests using a 1:1 mixed template of mutant and wild‐type sequences and analyzed the products by Sanger sequencing. We found that the repressive probe enabled efficient targeted amplification of 1607T>C, 1607T>G, 1609T>A, and 1610A>G mutations (Figure 4B–E).

### 3.4 Testing the Enrichment Efficiency of the Nucleic Acid Switch for Mutant Sequences

**Figure 5:**
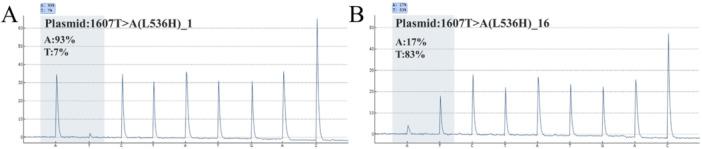
The repressive probe targeting ESR1 CDS 1607 shows strong amplification capability for low‐abundance mutations. (A) Amplification performance of the repressive probe in samples with high mutant allele frequency. (B) Amplification performance of the repressive probe in detecting low‐level, rare mutations.

To further evaluate the enrichment effect of the switch‐blocker on target region mutant sequences under different mutant‐to‐wild‐type ratios, we established a series of mixed templates with gradient‐diluted proportions of mutant and wild‐type sequences. Amplification was performed under conditions where the switch‐blocker inhibits wild‐type sequence amplification, followed by product analysis using pyrosequencing (Table 1). In the gradient dilution series for the L536H mutant, the highest pre‐amplification mutant allele fraction was 50%, which increased to 93% after amplification. The lowest pre‐amplification mutant allele fraction was 0.0015%, which increased to 16.89% post‐amplification (Table 1 and Figure 5A,B). In the E380Q mutation gradient dilution series, the highest input mutant allele fraction was 50%, which increased to 70.88% after amplification. At the lowest input level of 0.0015%, the mutant allele fraction reached 1.35% post‐amplification (Table 2).

**Table 1:** Pyrosequencing results of targeted amplification of L536H mutant templates at gradient dilution ratios.

A frequency (%)	Sample ID	A frequency (%)	C frequency (%)	G frequency (%)	T frequency (%)
50	1607T>A_1	93.13	—	—	6.87
25	1607T>A_2	80.73	—	—	19.27
12.5	1607T>A_3	69.64	—	—	30.36
6.25	1607T>A_4	57.14	—	—	42.86
3.125	1607T>A_5	41.74	—	—	58.26
1.5625	1607T>A_6	33.65	—	—	66.35
0.78125	1607T>A_7	22.57	—	—	77.43
0.390625	1607T>A_8	26.96	—	—	73.04
0.1953125	1607T>A_9	16.38	—	—	83.62
0.0976563	1607T>A_10	17.87	—	—	82.13
0.0488281	1607T>A_11	15.81	—	—	84.19
0.0244141	1607T>A_12	12.99	—	—	87.01
0.012207	1607T>A_13	14.85	—	—	85.15
0.0061035	1607T>A_14	20.3	—	—	79.7
0.0030518	1607T>A_15	19.1	—	—	80.9
0.0015259	1607T>A_16	16.89	—	—	83.11

**Table 2:** Pyrosequencing results of targeted amplification of E380Q mutant templates at gradient dilution ratios.

Frequency (%)	Sample ID	A frequency (%)	C frequency (%)	G frequency (%)	T frequency (%)
50	1138G>C_1	—	70.88	29.12	—
25	1138G>C_2	—	47.98	52.02	—
12.5	1138G>C_3	—	29.3	70.7	—
6.25	1138G>C_4	—	17.4	82.6	—
3.125	1138G>C_5	—	9.16	90.84	—
1.5625	1138G>C_6	—	5.21	94.79	—
0.78125	1138G>C_7	—	3.68	96.32	—
0.390625	1138G>C_8	—	2.88	97.12	—
0.1953125	1138G>C_9	—	2.32	97.68	—
0.0976563	1138G>C_10	—	1.49	98.51	—
0.0488281	1138 G>C_11	—	1.5	98.5	—
0.0244141	1138G>C_12	—	1.02	98.98	—
0.012207	1138G>C_13	—	1.55	98.45	—
0.0061035	1138G>C_14	—	0.91	99.09	—
0.0030518	1138G>C_15	—	1.12	98.88	—
0.0015259	1138G>C_16	—	1.35	98.65	—

This combination of mutation‐specific amplification using repressive probes and downstream pyrosequencing, which was validated across gradient‐diluted templates, showed the feasibility of the system and the strong mutant‐specific enrichment capacity of the repressive probes. Amplification of the L536H mutant series yielded stronger enrichment than that of the E380Q series. This discrepancy between mutant series may be attributed to factors such as the template concentration, probe concentration, and initial template ratio variation. Despite this finding, the E380Q‐targeting repressive probe still showed clear amplification capability, and further optimization is expected to improve its enrichment performance.

### 3.5 Applicability Testing of Switch‐Blockers in Clinical Samples

To verify the applicability of the switch‐blocker in clinical samples, we collected and processed plasma‐derived ctDNA from patients with breast cancer. The ctDNA was first analyzed using NGS, which identified 11 cases containing mutations at *ESR1* gene positions 1607–1613. We then performed targeted amplification of mutant sequences using the switch‐blocker, followed by product analysis via pyrosequencing. In the first round of amplification combined with pyrosequencing, the switch‐blocker–based targeted amplification system considerably enriched *ESR1* mutant sequences in patient‐derived ctDNA (Table 3).

**Table 3:** Comparison of next‐generation sequencing and pyrosequencing results before and after targeted amplification of circulating tumor DNA from clinical samples.

Sample ID	Next‐generation sequencing				(First time) pyrosequencing			
	Position	%	Position	%	Position	%	Position	%
1	1609TA	1.6			1609TA	10.69		
2	1610AC	16.6			1610AC	31.62		
3	1613AG	10.3			1613AG	36.15		
4	1610AC	5			1610AC	26.86		
5	1613AG	6.8			1613AG	15.67		
6	1613AG	7.1			1613AG	27.6		
7	1613AG	9.2			1613AG	36.58		
8	1609TA	1.2	1613AG	9	1609TA	28.81	1613AG	28.62
9	1609TA	31			1609TA	35.22		
10	1607TA	5.8			1607TA	43.12		
11	1610AC	1.1			1610AC	46.58		

## 4 Discussion

This study developed a combined novel technique for *ESR1* hotspot mutations through targeted amplification using switch‐blockers combined with pyrosequencing. In this study, a repressive probe specifically targeting the wild‐type sequence was rationally designed and optimized. This approach enabled selective amplification of the corresponding mutant sequences through an optimized PCR system based on the probe. Pyrosequencing was then used to analyze the PCR products derived from the selectively amplified mutant sequences, allowing for rapid detection of low‐abundance mutations. The proposed technical approach is based on circulating DNA obtained from liquid biopsies, which have garnered considerable attention in basic and clinical research because of their ability to comprehensively reflect the physiological status of patients. The developed method may help improve analytical sensitivity for selected *ESR1* mutations and offers a practical workflow for targeted analysis. This approach merits further clinical evaluation along with drug efficacy and patients' outcome data.

The transition to *ESR1* mutation‐directed therapies, such as next‐generation oral SERDs, requires companion diagnostics that are not only accurate but also accessible, scalable, and suitable for serial monitoring. Current gold standard methods face inherent trade‐offs. NGS provides breadth but at a cost and complexity that hinder routine, dynamic testing [28, 29, 30]. The ddPCR technique offers excellent sensitivity but is largely confined to known, predefined variants [31, 32, 33, 34]. Our pyrosequencing‐coupled selective amplification method addresses several of these issues. First, this amplification offers a balanced compromise because it achieves higher sensitivity than Sanger sequencing and can detect multiple mutation types in a single assay, unlike ddPCR which is typically limited to a small set of predefined variants per assay, while potentially maintaining lower operational cost and faster turnaround than comprehensive NGS. Second, the principle of selective enrichment enhances the signal from low‐abundance mutant alleles in a high background of wild‐type ctDNA, which is critical for the early detection of emerging resistance during adjuvant therapy or at the onset of progression. This enrichment may contribute to more reliable genotyping, which is fundamental for guiding the timely initiation of targeted therapies, such as elacestrant, in patients with acquired *ESR1* mutations [17, 18]. From a broader perspective, the design principle of our “nucleic acid switch”—using differential hybridization kinetics for allelic discrimination—is not limited to *ESR1*. Our approach provides a versatile blueprint for developing similar assays for other clinically actionable, low‐frequency hotspot mutations in genes, such as *PIK3CA*, potentially expanding the toolkit for affordable, multiplexed ctDNA monitoring across cancer types.

The clinical relevance of detecting *ESR1* mutations continues to expand. A recent study reported that ESR1 537 was associated with single‐nucleotide variants in the ER and RAF pathways and copy number variations in the MYC pathway and bone metastases, while ESR1 538 was associated with single‐nucleotide variants in the cell cycle pathway and liver metastases [35]. In the metastatic setting, pivotal trials, such as EMERALD, SERENA‐6, and EMBER‐3, have cemented the role of *ESR1* mutations as biomarkers for predicting a superior progression‐free survival benefit from novel oral SERDs compared with standard endocrine therapy [17, 18, 19]. Beyond monotherapy, understanding the co‐mutation landscape is crucial. The frequent co‐occurrence of *ESR1* and *PIK3CA* mutations presents a challenge and an opportunity. Co‐mutation may affect the therapeutic hierarchy and efficacy of combination strategies (e.g., SERD + phosphoinositide 3‐kinase inhibitor), indicating the need for comprehensive profiling that our multi‐variant assay begins to address [35].

Beyond the metastatic setting, the principles demonstrated by this assay indicate potential future applications in adjuvant care. Importantly, the following discussion represents a forward‐looking perspective based on the assay's technical performance, rather than conclusions drawn from the current dataset. In patients with breast cancer who experience recurrence after previous adjuvant AI therapy (including recurrence during adjuvant AI treatment), the *ESR1* mutation rate is approximately 4%–5% [36, 37, 38]. Emerging data have suggested that the detection of ctDNA during or after adjuvant AI therapy identifies a patient subset at exceptionally high risk for imminent distant recurrence [39, 40]. This finding creates a paradigm‐shifting opportunity for “molecular relapse” intervention. Based on the balance of sensitivity and cost‐effectiveness of our assay, it could hypothetically be evaluated in future studies as a tool for such longitudinal monitoring. The ability to detect and enrich these mutations from plasma could enable clinicians to identify patients with evidence of resistant clone expansion before radiographic recurrence occurs. This identification could lead to clinical trials and eventually practice, where adjuvant therapy is dynamically adapted (e.g., switching from an AI to a potent SERD or a SERD‐based combination upon detection of a rising *ESR1* mutant clone). However, we acknowledge that prospective clinical trials and real‐world data are essential prerequisites. This proactive, ctDNA‐guided intervention strategy represents the frontier of precision adjuvant therapy, aiming to eradicate micrometastatic resistant disease and improve long‐term cure rates.

This study had some technical limitations. Pyrosequencing is the gold standard method for precise quantitative analysis of known hotspot mutations in clinical settings. This method offers unique value for rapid, low‐cost, and highly sensitive detection of low‐frequency variants and methylation status. Therefore, in this technical approach, we attempted to combine the mutant sequence amplification system with pyrosequencing. This approach aimed to provide an economical, efficient, and highly sensitive mutation detection method for clinical practice. In certain mutation types, we confirmed the effectiveness of this combined approach, which can considerably amplify existing mutant “signals.” The pyrosequencing feature of simultaneously detecting base types within the sequenced region also greatly simplified the amplification and sequencing systems. Pyrosequencing showed strong application value for single known point mutations. However, pyrosequencing has an inherent technical limitation in that it cannot accurately sequence homopolymeric regions (multiple consecutive identical bases). This technology determines base types by detecting pyrophosphate released upon individual nucleotide incorporation, which is converted into a light signal. When consecutive identical bases (e.g., “AAAA” or “GGGG”) are present in the sequence, these bases are incorporated continuously in a single reaction cycle. The resulting light signal intensity should theoretically be proportional to the number of incorporated bases. However, in practice, the chemiluminescence signal intensity does not maintain a strict linear relationship with the base count. The accuracy of signal interpretation greatly declines, especially when the homopolymer length exceeds 4–5 bases. Beyond this inherent signal bias, quantification of base proportion in pyrosequencing is also affected by the interpretation of the software. An example of this interpretation is that, in quantifying a simple known point mutation percentage, the baseline is stable. Non‐mutant sites show 100% wild‐type peak intensity. Using these other stable wild‐type peaks as a calculation reference, the software can effectively perform data quality assessment and calculate the percentage of different bases at the mutant site. However, the *ESR1* mutations examined in this study involved multiple base changes at consecutive positions. This situation creates two computational challenges. First, there is a non‐linear relationship between fluorescent signal intensity and the number of consecutive identical bases. Second, there is a non‐standard “baseline” caused by multiple adjacent sites with varied base alterations.

In the technical implementation of this study, we observed that targeted adjustment of the nucleotide dispensation order during pyrosequencing, based on the wild‐type reference sequence, could improve the accuracy of software‐based signal interpretation and partially mitigate errors associated with reading consecutive identical bases to some extent. However, this optimization does not fundamentally overcome the intrinsic technical limitations of pyrosequencing. Accordingly, we recognize that the clinical application of switch‐blocker–assisted pyrosequencing for rapid mutation typing is subject to inherent limitations. Under appropriate sequence contexts, this combined strategy can achieve favorable detection performance. However, when mutations introduce extended homopolymeric stretches, particularly consecutive CCC or GGG motifs, the detection accuracy becomes suboptimal. Moving forward, translating this proof‐of‐concept assay into a scalable platform will require refining probe design strategies to minimize performance disparities between different mutation types.

## 5 Conclusion

In this study, we have developed a novel and practical selective amplification‐based assay for detecting *ESR1* hotspot mutations. This technology addresses key limitations of current sequencing methods by offering a sensitive, multiplex‐capable, and cost‐effective approach suitable for dynamic ctDNA monitoring. This technology is intended to serve as a feasible tool for guiding therapy in metastatic disease to enabling early detection of resistance in the adjuvant setting, where it holds the potential to trigger timely therapeutic interventions. As the field of precision oncology advances toward increasingly personalized and pre‐emptive treatment strategies, accessible and reliable tools for molecular monitoring similar to our approach will be valuable in improving outcomes for patients with ER+ breast cancer.

## Author Contributions

**Yantong Zhou:** methodology (lead); software (lead); formal analysis (lead); data curation (lead); investigation (lead); validation (lead); visualization (lead); writing – original draft (lead); writing – review and editing (equal). **Wenna Wang:** methodology (lead); investigation (equal); data curation (equal); resources (lead); writing – original draft (lead); writing – review and editing (lead). **Bo Lan:** methodology (supporting); investigation (supporting); data curation (supporting); resources (supporting); writing – original draft (supporting); writing – review and editing (supporting). **Chunxiao Li:** methodology (supporting); investigation (supporting); resources (supporting); writing – review and editing (supporting). **Jinsong Wang:** methodology (supporting); investigation (supporting); resources (supporting); writing – review and editing (supporting). **Ting Wang:** methodology (supporting); investigation (supporting); data curation (supporting); writing – review and editing (supporting). **Fangzhou Sun:** methodology (supporting); investigation (supporting); data curation (supporting); writing – review and editing (supporting).**Yan Wang:** conceptualization (lead); supervision (lead); resources (lead); funding acquisition (lead); project administration (lead); writing – review and editing (equal). **Haili Qian:** conceptualization (lead); supervision (lead); project administration (lead); resources (lead); methodology (equal); investigation (equal); data curation (equal); writing – original draft (equal); writing – review and editing (equal). **Fei Ma:** conceptualization (lead); supervision (lead); funding acquisition (lead); project administration (lead); resources (lead); writing – review and editing (equal).

## Ethics Statement

This study was approved by the Regulatory and Ethics Committees of National Cancer Center/National Clinical Research Center for Cancer/Cancer Hospital, Chinese Academy of Medical Sciences and Peking Union Medical College(approval number: 16‐038/1117).

## Consent

The authors have nothing to report.

## Conflicts of Interest

Professor Haili Qian and Fei Ma are members of the *Cancer Innovation* Editorial Board. To minimize bias, they were excluded from all editorial decision‐making related to the acceptance of this article for publication. The remaining authors declare no conflict of interest.

## Supporting information


**Supplement Table 1:** Enrichment of mutant copies in single‐mutation dilution samples with adjusted primer ratio.

## Data Availability

The data that support the findings of this study are available on request from the corresponding author. The data are not publicly available due to privacy or ethical restrictions.
