# Supplementary material for: A Novel Sensitive Technique to Detect ESR1 Hotspot Mutations in Liquid Biopsy Using Switch‐Blocker–Enhanced Targeted Amplification Coupled With Pyrosequencing
Source: Cancer Innov. 2026 Apr 6;5(2):e70054. doi: 10.1002/cai2.70054 (PMC13053169; doi:10.1002/cai2.70054)
Supplement: Supplementary file 1 — Supplement Table 1: Enrichment of mutant copies in single‐mutation dilution samples with adjusted primer ratio. [file CAI2-5-e70054-s001.doc]

Supplement table 1. Enrichment of mutant copies in single-mutation dilution samples with adjusted primer ratio.

| Sample | Sample type | Frequency (%) | Primer ratio | A Frequency (%) | C Frequency (%) | G Frequency (%) | T Frequency (%) | (Mean ± std) |
| --- | --- | --- | --- | --- | --- | --- | --- | --- |
| 1607T>A | plasmid | 50 | 1:2:5 | 80.72 | − | − | 19.28 | (80.99±1.82) |
| 1607T>A | plasmid | 50 | 1:5:5 | 78.58 | − | − | 21.42 |  |
| 1607T>A | plasmid | 50 | 1:5:10 | 82.83 | − | − | 17.17 |  |
| 1607T>A | plasmid | 50 | 1:10:10 | 81.83 | − | − | 18.17 |  |
| 1138G>C | plasmid | 50 | 1:2:5 | − | 62.79 | 37.21 | − | (63.73±2.47) |
| 1138G>C | plasmid | 50 | 1:5:5 | − | 61.22 | 38.78 | − |  |
| 1138G>C | plasmid | 50 | 1:5:10 | − | 67.07 | 32.93 | − |  |
| 1138G>C | plasmid | 50 | 1:10:10 | − | 63.85 | 36.15 | − |  |
| 1138G>C | ctDNA | 2 | 1:2:5 | − | 21 | 79 | − | (20.65±1.05) |
| 1138G>C | ctDNA | 2 | 1:5:5 | − | 20.73 | 79.27 | − |  |
| 1138G>C | ctDNA | 2 | 1:5:10 | − | 21.67 | 78.33 | − |  |
| 1138G>C | ctDNA | 2 | 1:10:10 | − | 19.19 | 80.81 | − |  |
